# Tillage Changes Vertical Distribution of Soil Bacterial and Fungal Communities

**DOI:** 10.3389/fmicb.2018.00699

**Published:** 2018-04-09

**Authors:** Ruibo Sun, Wenyan Li, Wenxu Dong, Yinping Tian, Chunsheng Hu, Binbin Liu

**Affiliations:** ^1^Key Laboratory of Agricultural Water Resources, Hebei Laboratory of Agricultural Water-Saving, Center for Agricultural Resources Research, Institute of Genetics and Developmental Biology, Chinese Academy of Sciences, Shijiazhuang, China; ^2^University of Chinese Academy of Sciences, Beijing, China

**Keywords:** conventional tillage, rotary tillage, no tillage, depth decay, soil bacterial community, soil fungal community

## Abstract

Tillage can strongly affect the long-term productivity of an agricultural system by altering the composition and spatial distribution of nutrients and microbial communities. The impact of tillage methods on the vertical distribution of soil microbial communities is not well understood, and the correlation between microbial communities and soil nutrients vertical distributions is also not clear. In the present study, we investigated the effects of conventional plowing tillage (CT: moldboard plowing), reduced tillage (RT: rotary tillage), and no tillage (NT) on the composition of bacterial and fungal communities within the soil profile (0–5, 5–10, 10–20, and 20–30 cm) using high-throughput sequencing of the microbial 16S/ITS gene. Microbial communities differed by soil properties and sampling depth. Tillage treatment strongly affected the microbial community structure and distribution by soil depth, and changed the vertical distribution of soil bacterial and fungal communities differently. Depth decay of bacterial communities was significantly smaller in CT than in RT and NT, and that of fungal communities were significantly greater in RT than CT and NT. The presence/absence of species was the main contributing factor for the vertical variation of bacterial communities, whereas for fungal communities the main factor was the difference in relative abundance of the species, suggesting niche-based process was more important for bacterial than fungal community in structuring the vertical distribution. Soil total carbon was correlated more with soil bacterial (especially the anaerobic and facultatively anaerobic groups) than with fungal community. These results suggested different roles of bacteria and fungi in carbon sequestration of crop residue and in shaping soil carbon distribution, which might impact on soil fertility.

## Introduction

Tillage practices can affect the physical, chemical, and biological properties of soil and have a strong impact on the health and productivity of agricultural ecosystems. Conventional plowing tillage (CT) has been used for centuries as the major method for seedbed preparation and weed control; however, it can cause severe erosion during the early cropping stage, which is considered the major environmental threat to sustainability and the productive capacity of agriculture (Pimentel et al., 1995; Montgomery, 2007). Alternative tillage practices such as reduced tillage (RT) or no tillage (NT), which create less erosion and runoff than CT (Lopez et al., 2000; Basic et al., 2004; He et al., 2011), are generally viewed as being more sustainable cultivation systems for the future (Hobbs et al., 2008). The key role of soil microbial communities in ecosystems has been brought to the forefront; therefore, many studies have focused on the impact of tillage on soil microbial communities and have found that conservation tillage techniques increase microbial abundance (or biomass) (Valpassos et al., 2001; Mathew et al., 2012; Guo et al., 2016), microbial diversity (Sheibani and Ahangar, 2013; Habig and Swanepoel, 2015), and enzyme activity (Nivelle et al., 2016; Zuber and Villamil, 2016).

Previous studies have also shown that the impact of different tillage techniques on microbial communities at different soil depths can vary. A study of tillage plots at seven US locations showed that aerobic microorganisms, facultative anaerobes, and denitrifiers were more abundant in the surface soils (0–7 cm) with NT than with CT; however, the trend reversed in the deeper layer soils (7–30 cm) (Doran, 1980). Another study in Ireland found that RT increased the total biomass of both bacteria and fungi in the 0–5 cm soil layer; however, it decreased the biomass of bacteria in the 5–20 cm soil layer (van Groenigen et al., 2010). It has been established that tillage methods could influence the vertical distribution of soil microbial communities; however, few studies have focused on the extent that the distribution of microbial communities within the soil profile has been altered under different tillage regimes. For this purpose, we developed a new concept called depth decay, in which the degrees of changes in both soil nutrients and microbial communities caused by different tillage methods could be assessed.

Tillage is frequently combined with crop residue retention, which is considered a way to improve soil quality and reduce the environmental effects caused by residue burning. It has been proven that crop residue incorporation increases soil carbon content, nutrients efficiency, and crop yield (He et al., 2009; Lu et al., 2009; Lehtinen et al., 2014; Reiter, 2015; Song et al., 2016; Chen et al., 2017). However, these effects are highly depth dependent, for example, Chen et al. (2017) found that nitrogen content under rotary tillage (RT) with straw returning was higher than that under deep plowing tillage with straw returning in the top soil layer (0–10 cm), but was lower in the layer below 20 cm. Under straw returning, plowing tillage resulted in lower total organic carbon and dissolved organic carbon in the 0–7 and 7–14 cm soil layers, respectively, but higher dissolved organic carbon in the 14–21 cm soil layer (Zhu et al., 2014). Activities of soil enzymes are also affected by tillage methods. It has been reported that RT with straw returning induced higher activities of sucrose, protease, and urease than deep plowing tillage in the 0–10 cm soil layer; however, lower activities were seen in the 10–20 and 20–30 cm soil layers (Chen et al., 2017). Oxygenis a key parameter influencing soil microbial activity and soil carbon and nitrogen cycling. Compared with NT, tillage could increase soil aeration porosity and oxygen diffusion rate (Khan, 1996). Hyperoxic conditions could increase the degradation of organic matters and cause higher carbon dioxide emission and lower carbon sequestration (Stęepniewski and Stęepniewska, 2009), which is closely correlated with soil microbial community, but the contribution of microbial groups with different preference for oxygen is still unclear (Keiluweit et al., 2017).

Although the impacts of tillage on soil microbes have been widely studied, the majority of these studies have been based on traditional techniques such as the chloroform fumigation-incubation method (Carter, 1986), direct counting of microorganisms (Norstadt and Mccalla, 1969; Linn and Doran, 1984), phospholipid fatty acid analysis (Sipilä et al., 2012), denaturing gradient gel electrophoresis analysis (Ibekwe et al., 2002; van Groenigen et al., 2010), and monitoring certain metabolic characteristics, including carbon utilization pattern (Guo et al., 2016) or respiration (Jiang et al., 2011). These studies have been unable to provide detailed and comprehensive phylogenetic or taxonomic information of the microbial communities, which is critically required when linking nutrient dynamics to functional microbial groups. In addition, the correlations between the vertical distribution of soil nutrients and soil microbial communities are still not clear. To bridge these gaps, in the present study bacterial and fungal communities in four soil layers (0–5, 5–10, 10–20, and 20–30 cm) under three tillage methods (conventional plowing tillage, CT; RT/rotary tillage, and NT) were studied using high-throughput sequencing techniques, and the relationship between the distributions of soil nutrients and microbial communities within the soil profile was analyzed.

The vertical distribution of bacterial communities has previously been reported as mainly being driven by deterministic processes related to soil pH, conductivity, and organic carbon content (Hu et al., 2015); therefore, we hypothesized that the tillage would decrease the vertical compositional variation of soil microbial communities via the homogenization of soil properties. We tested this hypothesis by inspecting the responses of bacterial and fungal communities to different tillage regimes, and the correlations between the vertical distributions of soil nutrients and microbial communities through linking the depth decay of soil physico-chemical parameters to that of the microbial community compositions.

## Materials and Methods

### Experiment Design

The site and design of experiments have been described in detail in a previous study (Dong et al., 2008). Briefly, the experiment was conducted in Luancheng County, Hebei province, China (37°53’ N, 114°41’ E), at an altitude of 50.1 m with typical fluvo-aquic soils. The experiment commenced in 1999 with three treatments that utilized different tillage methods: (1) CT: moldboard plowing, (2) RT, and (3) NT, with each treatment having three replicated plots. Different tillage practices were performed after the application of chopped corn residue on the soil surface after corn harvest in the October of each year. The cropping system of the experiment was wheat-maize rotation. Urea and diammonium hydrogen phosphate were used as nitrogen and phosphate fertilizer, respectively. Pre-plant fertilization was performed with 130 kg N ha^-1^ and 121 kg P ha^-1^ before winter wheat sowing. In addition, 138 kg N ha^-1^ was applied shortly after jointing stage of wheat and corn, respectively. All three treatments received identical fertilization, irrigation, and other agricultural management strategies.

### Soil Sampling and Chemical Analysis

Soil samples were collected on September 21, 2016. Soil from 0 to 30 cm was divided into four layers: 0–5, 5–10, 10–20, and 20–30 cm. Soil cores from each plot were collected from seven positions along a zigzag line (Carter, 1993), and the seven soil cores were mixed thoroughly to form a composite sample. Soils were sieved through 2 mm mesh screens to remove plant residues, stones, and other impurities, separated into two equal parts, and stored at 4 and -80°C for chemical analysis and DNA extraction, respectively.

Soil chemical characteristics were measured based on the methods described in a previous study (Chu and Grogan, 2010). Soil pH was measured in a soil:water ratio of 1:5 (weight/volume) using a pH meter (Mettler-Toledo FE28, Switzerland). Soil total carbon (TC) and total nitrogen (TN) were measured with a CHNOS elemental analyzer (Vario MAX, Elementar, Germany) after samples were ground and sieved through 150 μm mesh screens. Soil organic carbon (SOC) was determined using dichromate oxidation method (Shaw, 1959). Soil available potassium (AK) was extracted by ammonium acetate (1 M) and measured using a flame photometry (FP640, INASA Instrument, China). Soil available phosphorus (AP) was extracted by NaHCO_3_ (0.5 M) and measured using the molybdenum blue method (Crouch and Malmstadt, 1967).

### DNA Extraction, PCR, and High-Throughput Sequencing

Soil total DNA was extracted using a Fast^®^DNA SPIN Kit (MP Biomedicals, Santa Ana, CA, United States) and applying the protocol described in the manual. Primer sets 515F/806R (Caporaso et al., 2011) and ITS3/ITS4 (Orgiazzi et al., 2012) were used to amplify the V4 region of the bacterial 16S rRNA gene and region 2 of the fugal internal transcribed spacer (ITS). PCRs were performed in a 50-μL mixture containing 25 μL PCR premix (TaKaRa Ex Taq^®^), 1 μL forward primer (10 μM), 1 μL reverse primer (10 μM), 1 μL DNA template (20 ng), and 22 μL PCR-grade water under the following conditions: initial denaturation at 94°C for 10 min, 30 cycles in a series of denaturation at 94°C for 1 min, annealing at 50 (for bacteria) or 56°C (for fungi) for 1 min, and extension at 72°C for 1 min, then a final extension at 72°C for 10 min. The product of PCR was visually checked by agarose gel electrophoresis and then purified with AMPure XP beads (Beckman Coulter Inc., Brea, CA, United States). Illumina sequencing adapters and dual-index barcodes were added through a subsequent eight-cycle PCR for library preparation and sample distinguishing, respectively. PCR products were then sequenced using a Illumina HiSeq 2500 system following the methods described in the manuals of the library preparation and sequencing kits (Caporaso et al., 2012). The raw data were deposited in the European Nucleotide Archive under accession number PRJEB21463.

### Analysis of the High-Throughput Sequencing Data

The high-throughput sequencing data were analyzed with the bioinformatics pipeline of Quantitative Insights Into Microbial Ecology (QIIME) (version 1.9.1) (Caporaso et al., 2010). The adaptor sequence, barcode, and 30 low-quality bases at the end of each read were cut off, and then the forward and reverse Illumina reads were joined using the fastq-join method (Aronesty, 2011) with minimum overlap of 20 bp and maximum allowed 10% mismatches within overlap region. Sequences with Phred quality score <20 or length shorter than 200 bp were discarded. Chimeras of bacterial and fungal sequences were removed by the UCHIME algorithm (Edgar et al., 2011) in the USEARCH package (Edgar, 2010) against the “Gold” database and UNITE fungal ITS reference data set (version 7.0) (Nilsson et al., 2015), respectively. The clean data were then clustered into operational taxonomic units (OTUs) at 97% similarity using UCLUST method (Edgar, 2010). The longest sequence within each OTU was selected as the representative sequence. Taxonomic assignment of each OTU was conducted using RDP Classifier (Wang et al., 2007) with a minimum confidence of 0.80. Singletons and OTUs not assigned as bacteria or fungi were filtered out, and the bacterial and fungal OTU tables were subsampled to 14,000 and 24,000 sequences per sample, respectively, for further analysis. Phenotypes of bacterial communities were predicted using BugBase (Ward et al., unpublished).

### Statistical Analysis

Multiple co-inertia analysis (MCIA) (Meng et al., 2014) was performed to identify the changing trends of microbial communities in different tillage treatments and soil layers. MCIA was implemented using the omicade4 package (Meng et al., 2014) in R (version 3.4.0). Heatmaps and Venn diagrams were drawn using the gplots library in R to show the distribution of microbial communities in different soil depths and tillage regimes. The contribution of OTUs to the dissimilarity of community between treatments was calculated using similarity percentage (SIMPER) analysis in the vegan package in R (Clarke, 1993). To evaluate the degree of changes in the soil characteristics and microbial communities within the vertical soil profile caused by different tillage regimes, we developed a new concept called depth decay, which was obtained by linear regressions between microbial community/soil nutrients similarity and depth distance. The depth decays of the soil TC, TN, and microbial communities of bacteria and fungi were calculated using linear regressions. Differences of the slopes were checked using the diffslope function in the simba library (Nekola and White, 1999) of R based on 10,000 permutations. Redundancy analysis (RDA) and the Mantel test were applied to determine the correlations between soil properties and microbial communities, which were performed using the vegan library in R. Function envfit was used for selected environmental variables. Environmental variables that were not significantly correlated (*P* > 0.05) with the RDA model were removed. Pearson correlations between environmental variables and microbial communities (Bray–Curtis distance) were calculated by the Mantel test. The relative contribution of soil depth and tillage to the variance of soil properties, bacterial, and fungal communities was determined by canonical variation partitioning (Borcard et al., 1992). Automatic linear modeling was performed to identify the relative importance of different bacterial groups for soil TC at 95% confidence level in IBM SPSS Statistics for Windows (Sun et al., 2015). The Kruskal–Wallis test (Corder and Foreman, 2009) was used to assess the significance of differences of variables between different groups. The abundance-based β-null deviation was calculated to differentiate niche and neutral process for bacterial and fungal community assemble (Tucker et al., 2016).

## Results

### Variation of Soil Properties

The majority of soil properties varied within the different soil layers (**Table 1**). Soil pH increased with soil depth, and soil TC, TN, SOC, and AK decreased from the shallow layer to the deep layer. The vertical distribution of soil properties was impacted by tillage methods. Soil pH of the 5–10 cm layer under RT was significantly lower than that under CT and NT; however, it was much higher in the 20–30 cm layer. The NT treatment had the highest soil TC in the top two layers (0–5 and 10–20 cm), whereas the lowest TC in the 20–30 cm layer was observed in the RT treatment. The NT treatment resulted in the highest TN within the four layers, and there was no significant difference between the CT and RT treatments. Soil SOC was highest in NT treatment at the top two layers and varied little among the three treatments in the two lower layers. Soil electric conductivity, C:N ratio, and content of AK and AP varied little under different tillage treatments. Significant changes in C:N were only observed in the 10–20 cm layer, in which NT had lower C:N than CT and RT. Significantly lower content of AK in the NT treatment was only observed in the 20–30 cm layer. RT resulted in a significantly lower AP content in the 0–5 cm layer and NT had higher AP in the 10–20 cm layer. In summary, the different effects of tillage practices on soil properties within different soil layers changed the vertical distribution, and the vertical variation in soil properties was lower in the CT and RT treatments than in the NT treatment (Supplementary Figure S1). By variation partitioning, the contribution of soil depth to the variance of soil properties was much higher than that of tillage (Supplementary Table S3), but tillage practices (CT and RT) reduced the vertical heterogeneity of soil properties (Supplementary Figures S1, S5 and Table S2).

**Table 1:** Soil characteristics in different tillage regimes and soil layers.

Properties	Depth (cm)	Treatment		
		CT	RT	NT
pH	0–5	7.43 ± 0.03a^†^	7.41 ± 0.03a	7.46 ± 0.04a
	5–10	7.46 ± 0.00a	7.43 ± 0.01b	7.46 ± 0.04ab
	10–20	7.49 ± 0.02a	7.48 ± 0.03a	7.49 ± 0.02a
	20–30	7.63 ± 0.07b	7.76 ± 0.01a	7.66 ± 0.05b
EC (μS cm^-1^)	0–5	174 ± 5b	195 ± 27ab	234 ± 59a
	5–10	186 ± 20a	169 ± 6a	186 ± 6a
	10–20	158 ± 5a	166 ± 10a	201 ± 55a
	20–30	183 ± 24ab	200 ± 22a	166 ± 13b
TC (%)	0–5	1.84 ± 0.06b	1.99 ± 0.16ab	2.11 ± 0.08a
	5–10	1.76 ± 0.02b	1.78 ± 0.03ab	1.85 ± 0.09a
	10–20	1.58 ± 0.03a	1.53 ± 0.00b	1.53 ± 0.06ab
	20–30	1.39 ± 0.05a	1.19 ± 0.02b	1.35 ± 0.06a
TN (%)	0–5	0.175 ± 0.005b	0.183 ± 0.007b	0.206 ± 0.005a
	5–10	0.166 ± 0.003b	0.169 ± 0.013ab	0.185 ± 0.017a
	10–20	0.145 ± 0.007ab	0.136 ± 0.009b	0.162 ± 0.017a
	20–30	0.118 ± 0.005a	0.100 ± 0.009b	0.128 ± 0.021a
C:N	0–5	10.5 ± 0.5a	10.9 ± 1.1a	10.3 ± 0.5a
	5–10	10.6 ± 0.3a	10.6 ± 0.7a	10.0 ± 0.5a
	10–20	10.9 ± 0.8a	11.3 ± 0.7a	9.48 ± 0.58b
	20–30	11.8 ± 0.5a	12.0 ± 0.9a	10.8 ± 1.4a
SOC (%)	0–5	1.72 ± 0.01b	1.65 ± 0.06c	1.82 ± 0.14a
	5–10	1.39 ± 0.04b	1.52 ± 0.16ab	1.61 ± 0.12a
	10–20	1.25 ± 0.03a	1.16 ± 0.05b	1.28 ± 0.09a
	20–30	0.97 ± 0.08a	0.93 ± 0.03a	0.94 ± 0.01a
AK (mg kg^-1^)	0–5	157 ± 29a	130 ± 8a	143 ± 9a
	5–10	100 ± 8a	100 ± 8a	100 ± 8a
	10–20	83 ± 5a	83 ± 5a	77 ± 5a
	20–30	80 ± 0a	77 ± 5a	70 ± 0b
AP (mg kg^-1^)	0–5	20.2 ± 6.0ab	16.0 ± 2.8b	22.7 ± 5.7a
	5–10	10.3 ± 0.9a	15.3 ± 6.1a	9.16 ± 3.0a
	10–20	9.44 ± 1.65b	13.7 ± 3.0ab	15.9 ± 4.9a
	20–30	10.7 ± 4.4a	14.3 ± 1.0a	12.3 ± 2.6a

*^†^Means followed by the same letter within the same soil layer are not significantly different by two-tailed Kruskal–Wallis test at *P* < 0.05. EC, electric conductivity; TC, total carbon; TN, total nitrogen; SOC, soil organic carbon; AK, available potassium; AP, available phosphorus.*

### Bacterial and Fungal Community Composition Under Different Tillage Methods

**FIGURE 1:**
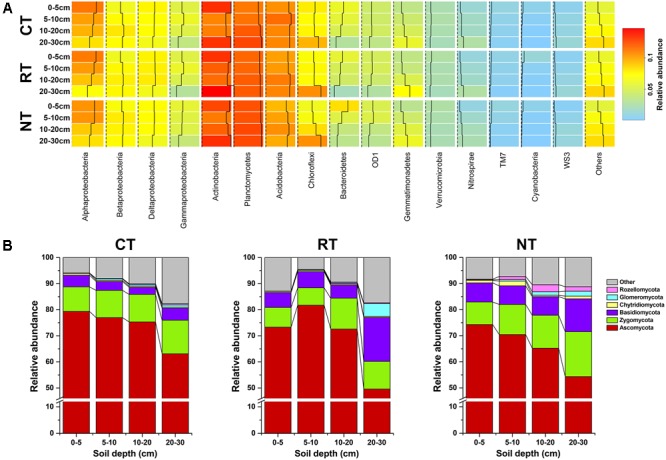
Community composition of soil bacteria **(A)** and fungi **(B)** in different soil depths under three tillage regimes. CT, conventional plowing tillage; RT, rotary tillage; NT, no tillage.

After quality filtering, 797666 bacterial 16S rRNA gene and 1275096 fugal ITS2 clean reads were obtained, which clustered into 45,853 and 8084 OTUs, respectively. The bacterial community was composed of 15 dominant phyla (subphyla) with relative abundance >1% (**Figure 1A**). Proteobacteria, Actinobacteria, Planctomycetes, and Acidobacteria were the four most dominant phyla, which accounted for 30.57, 12.42, 12.0, and 10.49% of the total reads, respectively. The ITS sequences were assigned to six phyla, and Ascomycota was the dominant phyla, which accounted for 69.69% of the total reads (**Figure 1B**).

**FIGURE 2:**
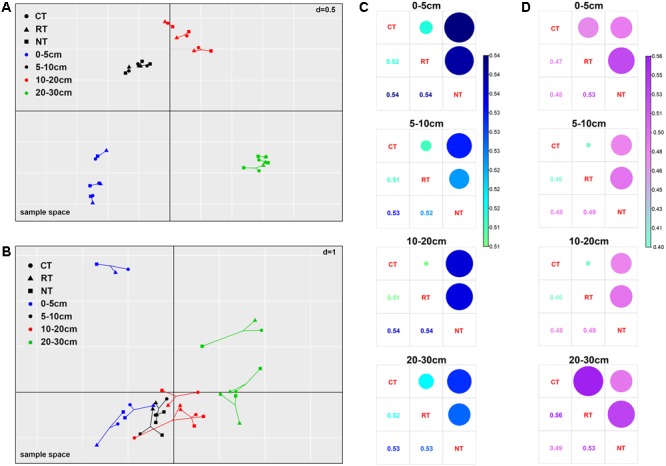
Multiple co-inertia analysis (MCIA) of the bacterial **(A)** and fungal **(B)** communities and Bray–Curtis dissimilarity between different tillage regimes of bacterial **(C)** and fungal **(D)** communities. CT, conventional plowing tillage; RT, rotary tillage; NT, no tillage.

The MCIA plot revealed the separation of soil bacteria within soil layers (**Figure 2A**). Samples from the same soil depth but different treatments were linked by edges. The short edges expressed high similarity of samples. Furthermore, the bacterial community of samples under different tillage regimes from the same soil layer were closely projected, which indicated high homogeneity in the bacterial community of samples from the same soil layer. This was also confirmed from the heatmap of soil bacterial community composition (**Figure 1A**). For example, the relative abundance of Alphaproteobacteria and Bacteroidetes decreased while that of Planctomycetes, Gemmatimonadetes, and Nitrospirae increased when soil depth increased (**Figure 1A**). Similar results of the dominant bacterial OTUs were also observed (Supplementary Figure S2A). Different from the bacterial community, the edges of the soil fungal communities within the same soil layer under different tillage regimes were longer in the MCIA plot (**Figure 2B**), indicating low similarity of different tillage regimes. In addition, samples from different soil layers were not clearly separated from each other. The distribution patterns of the dominant fungal OTUs within the soil profile were dramatically different under different tillage regimes (Supplementary Figure S2B).

The changes in bacterial and fungal communities under different tillage regimes were assessed by calculating the Bray–Curtis dissimilarity (**Figures 2C,D**). Dissimilarity of bacterial communities between CT and RT was lower than that between NT and CT, and NT and RT. This was observed in all four soil layers (**Figure 2C**). For the fungal community, similar results were observed in the top three soil layers; however, the largest dissimilarity was observed between CT and RT in the 20–30 cm soil layer (**Figure 2D**). The OTUs that changed significantly in relative abundance between different tillage regimes accounted for 14.6–16.9% of the total bacterial community, and the proportion was much higher in the fungal community, which ranged from 35.1 to 57.9%. In addition, the variance of fungal OTUs between tillage regimes was much higher than that of bacterial OTUs (Supplementary Figure S3). These results indicate that long-term tillage exerted greater effects on the fungal community than on the bacterial community.

### Bacterial and Fungal α-Diversity

Operational taxonomic unit richness and equitability (Shannon diversity divided by the logarithm of the number of taxa) were calculated to compare bacterial and fungal α-diversity among samples (**Table 2**). In the CT treatment, the highest bacterial richness was observed in the 5–10 cm layer, whereas the top layer (0–5 cm) had the highest bacterial richness in the RT treatment. In comparison, the bacterial richness in NT treatment was significantly lower in the surface soil layers (0–5 and 5–10 cm). Soils under the CT treatment had the highest fungal richness among the three tillage regimes, and NT resulted in lower fugal richness (**Table 2**). There were no significant changes in bacterial and fungal evenness among the three tillage regimes, except that NT had significantly higher fungal evenness in the 5–10 cm layer, followed by CT and RT.

**Table 2:** Richness and evenness of bacterial and fungal community in different tillage regimes and soil layers.

	OTU richness			Equitability		
	CT	RT	NT	CT	RT	NT
Bacteria						
0–5 cm	4633 ± 39b^†^	4720 ± 24a	4565 ± 68c	0.934 ± 0.001a	0.935 ± 0.001a	0.934 ± 0.002a
5–10 cm	4667 ± 64a	4626 ± 38a	4565 ± 9b	0.933 ± 0.001a	0.933 ± 0.001a	0.932 ± 0.001a
10–20 cm	4622 ± 81a	4607 ± 103a	4523 ± 129a	0.933 ± 0.001a	0.932 ± 0.002a	0.928 ± 0.005a
20–30 cm	4501 ± 76a	4437 ± 55a	4561 ± 110a	0.927 ± 0.003a	0.925 ± 0.002a	0.929 ± 0.002a
Average	4605 ± 92a	4597 ± 120a	4554 ± 93a	0.932 ± 0.003a	0.931 ± 0.004a	0.931 ± 0.004a
Fungi						
0–5 cm	1560 ± 74a	1375 ± 148ab	1322 ± 68b	0.650 ± 0.009a	0.601 ± 0.046a	0.672 ± 0.013a
5–10 cm	1467 ± 74a	1435 ± 32a	1293 ± 22b	0.643 ± 0.020b	0.605 ± 0.009c	0.679 ± 0.008a
10–20 cm	1387 ± 146ab	1530 ± 48a	1244 ± 32b	0.614 ± 0.059a	0.654 ± 0.027a	0.688 ± 0.026a
20–30 cm	1405 ± 35a	1112 ± 19b	1143 ± 66b	0.678 ± 0.005a	0.633 ± 0.054a	0.700 ± 0.022a
Average	1455 ± 114a	1363 ± 175a	1251 ± 85b	0.646 ± 0.039a	0.623 ± 0.044a	0.685 ± 0.022a

*^†^Means followed by the same letter in the same row are not significantly different at *P* < 0.05 by two-tailed Kruskal–Wallis test.*

### Vertical Distribution of Bacterial and Fungal Communities Under Different Tillage Regimes

**FIGURE 3:**
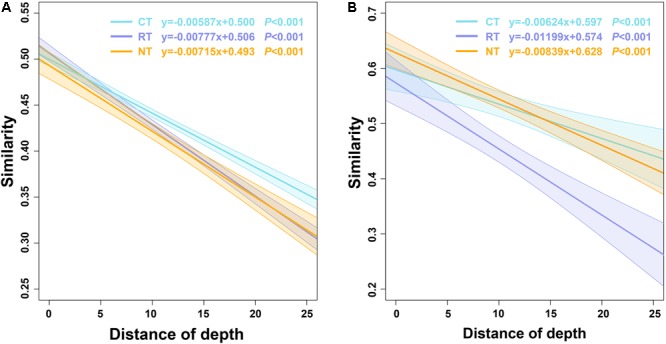
The depth decay of Bray–Curtis similarity for bacterial **(A)** and fungal **(B)** communities under different tillage practices. CT, conventional plowing tillage; RT, rotary tillage; NT, no tillage.

Significant negative relationships between microbial community similarity and vertical depth variation (depth decay) were observed in all three tillage regimes; however, the slopes varied (**Figure 3**). The slope of the RT treatment was higher than the NT treatment; however, the slope of the CT treatment was lower than the NT treatment, indicating the different tillage methods affected microbial vertical spatial variations differently. CT decreased the dissimilarity of the microbial community within soil layers, whereas RT increased the vertical spatial variations of the soil microbial community. However, the significance test revealed that the slope of the bacterial community in the CT treatment was significantly different from the RT and NT treatments, whereas RT and NT had no significant differences (Supplementary Table S1), indicating that CT dramatically homogenized the composition of the bacterial community. Conversely, the slope of the fungal community in the RT treatment was significantly different from the CT and NT treatments, with no significant differences between the CT and NT treatments. These results indicated that, compared to NT, RT had less influence on soil bacterial vertical variations than CT did; however, it had greater influence on soil fungal vertical variations.

**FIGURE 4:**
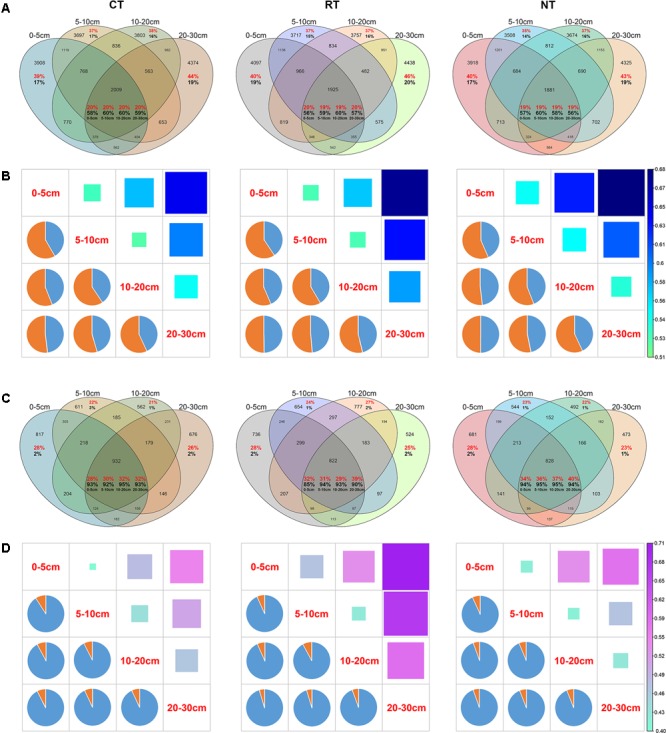
Venn diagram showing the distribution of bacterial **(A)** and fungal **(C)** OTUs in different soil layers under three tillage regimes. The red percentage shows the proportion of the OTU counts in the total number of OTUs. The black percentage shows the sum of the relative abundance of the OTUs. Heatmaps (squares on the upper right) and pie charts (left bottom) show the dissimilarity and the contribution of shared OTUs and unique OTUs to the dissimilarity between different soil layers, respectively, for bacterial **(B)** and fungal **(D)** communities. CT, conventional plowing tillage; RT: rotary tillage; NT: no tillage.

The distribution of bacterial and fungal OTUs within different soil layers was also analyzed to compare the different vertical distributions of bacterial and fungal species under the three tillage regimes (**Figures 4A,C**). Under the CT treatment, 3908 OTUs were only detected in the 0–5 cm soil layer, which accounted for 39% of the total OTUs and 17% of the bacterial community in this layer. In total, 2009 OTUs were shared across all four soil layers, accounting for approximately 20% of the total OTUs and 58–60% of the bacterial community in the four soil layers (**Figure 3A**). These ratios were similar in the RT and NT treatments for the bacterial community. Different from the bacterial community, the ratio and relative abundances of shared OTUs in the fungal community were much higher, accounting for 28–40% of total OTUs and 85–95% of relative abundance (**Figure 4C**). In addition, the ratios of unique OTUs in certain soil layers and the proportion in the entire fungal community were much lower (from 21 to 28% and 1 to 3%, respectively) (**Figure 4C**).

Dissimilarity of bacterial and fungal communities between soil layers was impacted by soil tillage (**Figures 4B,D**). The dissimilarity between bacterial and fungal communities became larger as the vertical distance increased. However, CT decreased the vertical dissimilarity of the bacterial community and RT increased the vertical dissimilarity of the fungal community (**Figures 4B,D** and Supplementary Figure S4), which indicated that CT homogenized the bacterial community and RT heterogenized the fungal community.

The contribution of shared OTUs and non-shared OTUs (not detected in all soil layers) was discriminated using SIMPER analysis (**Figures 4B,D**). Results showed that the shared OTUs contributed <50% of the dissimilarity of the bacterial community between different soil layers (**Figure 4B**). On the contrary, shared OTUs contributed >90% of the fungal vertical variation (**Figure 4D**). This indicated that the presence/absence of bacterial species played a greater role in the vertical variation of the bacterial community than of the fungal community. The changes in relative abundance of fungal species contributed most of the vertical variation of the fungal community.

### Correlations Between Soil Factors and Bacterial and Fungal Communities

**FIGURE 5:**
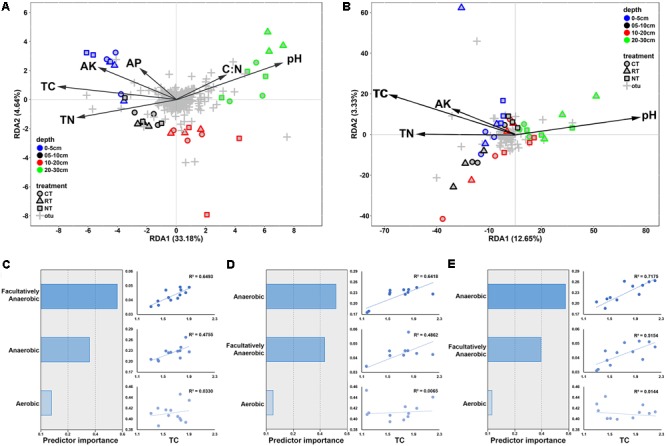
Redundancy analysis plots presenting the correlations between soil characteristics and bacterial **(A)** and fungal **(B)** community structures; predictive importance of anaerobic, aerobic, facultatively anaerobic bacteria to TC and linear regression between anaerobic, aerobic, facultatively anaerobic bacteria, and TC under CT **(C)**, RT **(D)**, and NT **(E)**. CT, conventional plowing tillage; RT, rotary tillage; NT, no tillage; TC, total carbon; TN, total nitrogen; AK, available potassium; AP, available phosphorus.

Redundancy analysis and the Mantel test were applied to determine correlations between soil factors and microbial communities. RDA explained 46.3 and 20.2% of the total variation in the soil bacterial and fungal community structures, respectively. Soil bacterial communities were distributed along the first axis from the surface layer to the deep layer on the RDA plot (**Figure 5A**). TC, TN, and pH were the top three factors correlated with bacterial and fungal communities (**Figure 5**). The results of the Mantel test also revealed that both bacterial and fungal communities were highly correlated with TC, followed by TN and pH (**Table 3**); however, higher correlations were observed between bacterial communities than fungal communities. Linear regressions between TC/TN similarity and depth distance were plotted to reveal the level of changes caused by different tillage regimes (Supplementary Figure S5). CT lowered the vertical spatial variance of TC and TN, whereas RT increased the vertical spatial variance of TC and TN. This relationship was consistent with the soil bacterial vertical depth decay pattern (**Figure 3A**), indicating that the vertical distribution of the soil bacterial community was closely correlated with the soil carbon and nitrogen vertical distribution.

**Table 3:** Correlations between soil microbial community and soil physico-chemical properties.

Bacterial community			Fungal community		
Factors	*r*	*P*-value	Factors	*r*	*P*-value
TC	0.860	0.001	TC	0.538	0.001
SOC	0.812	0.001	pH	0.529	0.001
TN	0.765	0.001	TN	0.449	0.001
pH	0.658	0.001	SOC	0.381	0.001
AK	0.560	0.001	AK	0.247	0.019
C:N	0.186	0.004	C:N	0.156	0.043
AP	0.109	0.088	EC	0.008	0.410
EC	0.098	0.132	AP	-0.044	0.641

*Mantel test was performed using “Pearson” correlation method. Similarity matrices of soil characteristics and microbial community structures were calculated based on Euclidean distance and Bray–Curtis distance, respectively. TC, total carbon; TN, total nitrogen; SOC, soil organic carbon; AK, available potassium; AP, available phosphorus; EC, electric conductivity.*

The biological interpretable phenotypes of the bacterial community were predicted using BugBase, and the phenotypes classified by oxygen tolerance (aerobic, anaerobic, and facultatively anaerobic bacteria) varied along soil depth and effected by tillage practice (Supplementary Figure S6). In order to determine the relative contribution of these groups to soil carbon sequestration, their correlations between soil TC were analyzed (**Figures 5C–E**). Anaerobic bacteria showed the highest relative importance for TC variance in RT and NT soil, followed by facultatively anaerobic bacteria. Facultatively anaerobic group was the most important predictor in CT soil. Linear regression between soil TC and the relative abundance of the phylotypes also revealed the highest correlation between TC and anaerobic bacteria in RT and NT, and between TC and facultatively anaerobic bacteria in CT.

## Discussion

Soil microbes play an important role in soil ecosystems and they are considered an indicator of soil quality (Sharma et al., 2011). However, their communities are easily disturbed by intensive agricultural practices (Girvan et al., 2004; Mueller et al., 2015; Sun et al., 2015, 2016). In the present study, we found that tillage practices changed the vertical distribution of soil bacterial and fungal communities, and soil bacterial and fungal communities responded differently to agricultural tillage. CT decreased the vertical spatial decay velocity of bacterial and fungal communities (**Figure 3**), indicating the homogenization of CT on soil microbial communities, which verified our initial hypothesis. CT had its most significant impact on the soil bacterial community, whereas RT had a greater impact on the soil fungal community (**Figure 3** and Supplementary Table S1). Furthermore, the vertical change in the fungal community was mostly due to changes in the relative abundance of fungal species, rather than changes in presence/absence. By contrast, for the soil bacterial community the presence/absence of bacterial species also played a key role in the bacterial vertical variation (**Figure 4**). Due to higher diversity of bacteria than fungi in soil, higher proportion of bacterial than fungal species with low abundance may not be covered under current sequencing effort, increasing sequencing depth may result in more accurate estimation of the contribution of the abundance and presence/absence to the vertical change. However, based on the current sequencing depth, our results indicated that niche filtering had a greater effect on the survival of soil bacteria than fungi. Bacterial cells are generally smaller than fungal cells and are more easily affected by the soil microenvironment (Mueller et al., 2015). By contrast, most fungi grow as hyphae, which can be up to several centimeters in length. In addition, fungi can produce spores, which are an effective dispersal stage and can travel through air or water, conferring greater mobility of fungi than bacteria. Thus, fungi cover a broader range of vertical habitats than bacteria do and soil bacterial communities are more sensitive to environmental changes or agricultural practices (Girvan et al., 2004; Barnard et al., 2013; Zhang et al., 2015). In the present study, we revealed that the bacterial community was more greatly impacted by soil depth than the fungal community was. This was further confirmed by the higher contribution of soil depth than tillage to bacterial community variance than that to fungal community (Supplementary Table S3).

The results of the present study also revealed the different contribution of niche-based processes and stochastic-neutral processes in structuring the vertical distribution of bacterial and fungal communities. The relative roles of niche and neutral processes in structuring soil microbial communities have mainly been previously studied to explain the horizontal distribution of soil microbial communities (Dumbrell et al., 2010; Nemergut et al., 2013; Kivlin et al., 2014; Liao et al., 2016), with few studies have focused on the vertical distribution of microbial communities (Hu et al., 2015). The present study provided some evidence to address this gap. The plowing practice creates homogenization of the soil, and if the soil microbial communities are shaped by the stochastic-neutral processes, then the microbial communities in different soil layers would be similar to each other after homogenization. However, this was not the case in the present study. Soil bacterial communities within different soil layers showed obvious separation from each other after 1 year of the nearest plowing (**Figures 2A, 5A**), and the vertical compositional variation of soil bacterial community was accordant to the changes of soil properties (**Figure 3A** and Supplementary Figure S5), indicating the great niche filtering effect of soil bacterial communities. By contrast, the distribution of fungal communities within soil depth was not as clear as bacterial communities (**Figures 2B, 5B**), and the vertical compositional variation of fungal community and the changes of soil properties were inconsistent (**Figure 3B** and Supplementary Figure S5). These results illustrated niche-based processes play more important role in structuring the vertical distribution of bacterial community than fungal community, which was further supported by the higher β-null deviation values for bacterial than fungal community (Supplementary Table S4). However, at the same time, the relative abundance of fungal OTUs varied in different soil layers and was influenced by tillage methods (Supplementary Figure S2B), indicating “niche-based” mechanisms (Tokeshi, 1990) also regulated fungal community assembly. In a previous study of fungal biogeography, both niche and neutral processes structured the arbuscular mycorrhizal fungal community (Dumbrell et al., 2010), and, at regional scales, environmental filtering had greater effects on soil fungal community composition than dispersal limitation (Kivlin et al., 2014). Results from the present study extend the research of niche and neutral processes in structuring fungal communities – both niche and neutral processes structured the vertical distribution of the fungal community.

The greater contribution of niche-based processes in structuring the vertical distribution of bacterial communities than of fungal communities was also supported by the higher correlation between soil properties and the bacterial community than the fungal community (**Figures 5A,B**), and the higher contribution of soil properties in shaping bacterial community than fungal community (**Table 3**). Both bacterial and fungal communities were mostly correlated with soil TC content; however, soil TC was correlated more with the soil bacterial community than with the fungal community, which indicated bacteria might play a greater role in shaping soil carbon vertical distribution. Carbon is one of the most important components for soil quality and its turnover is closely related to soil bacteria and fungi (Malik et al., 2016; Fabian et al., 2017). However, because of the unique physiologies and differential interactions with soil physical properties of bacteria and fungi, they play different roles in soil carbon cycling and storage (Six et al., 2006; Waring et al., 2013). Heterotrophic bacteria and fungi dominate microbial decomposer communities in soils and they have some functional overlap in decomposition. However, fungi have wider enzymatic capabilities and higher capacity for the decomposition of plant polymers than bacteria do. Thus, it is considered that fungi provide bacteria with resources that the bacteria are not able to acquire on their own (Romani et al., 2006). Studies have revealed that fungi and bacteria targeted different substrates during the degradation of organic matters (Tardy et al., 2015; Mora-Gómez et al., 2016). It has been revealed that, in aquatic systems, fungi are the forerunner for leaf letter decomposition, preparing the leaf litter for the microbial loop, whereas bacteria determine the amount of terrigenous carbon to be potentially recycled into the terrestrial environment via CO_2_ emission (Krauss et al., 2011; Fabian et al., 2017). Bacterial respiration was greatly determined by soil oxygen, and studies have revealed that tillage could increase soil aeration porosity (Khan, 1996), thus increase soil oxygen content to enhance the soil respiration and CO_2_ emission (Bridgham and Richardson, 1992; Schlesinger and Andrews, 2000; Stęepniewski and Stęepniewska, 2009). In this study, we found high correlations between carbon vertical distribution and anaerobic/facultatively anaerobic rather than aerobic bacteria (**Figures 5C–E**). This was supported by a recent study from Keiluweit et al. (2017), who revealed that the anaerobic soil microsites could selectively protect organic compounds from decomposition and therefore play an important role in soil carbon stabilization. A previous study using the soils from the same experiment discovered that the TC input from crop residue was highest in the CT treatment, following the RT and NT treatments, and the emission of CO_2_ was of the same order (Dong et al., 2008); however, soil TC content was the opposite, indicating higher carbon sequestration efficiency of the NT treatment. The results from this study suggested that the higher carbon sequestration efficiency in the NT treatment may be due to the change of anaerobic bacteria. The mechanisms of this phenomenon may link to the lower mineralization rate and carbon-use efficiency of anaerobic bacteria (Wania et al., 2009; Allison et al., 2010). Taken together, we speculate that the variation of soil carbon vertical distribution caused by tillage was mainly driven by the changes of anaerobic/facultatively anaerobic bacteria; however, the changes in soil fungal communities may also impact substrates used by soil bacteria by changing intermediate decomposition products of crop straw, thus resulting in different CO_2_ emission and carbon sequestration. However, NT led to a decrease in soil fungal richness (**Table 2**), with further study required to determine how this influences the decomposition of crop residue and the interactions between bacterial and fungal communities.

## Conclusion

Tillage methods significantly affected soil properties, with NT resulting in higher TC content than RT and CT. The soil fungal community was more responsive to soil tillage than the bacterial community; however, bacterial communities represented a clearer vertical spatial differentiation with soil depth than fungal communities because soil fungi covered a broader range of vertical habitats than that of bacteria. Soil bacterial richness was only slightly impacted by tillage methods, whereas fungal richness was significantly lower in NT than in RT and CT. Soil TC content was correlated more to the soil bacterial community (anaerobic/facultatively anaerobic bacteria) than to the fungal community. The present study suggested that different tillage methods influenced carbon sequestration of crop residue and the vertical distribution of soil carbon by changing the vertical distribution of soil fungal and bacterial communities.

## Author Contributions

RS, WL, and BL designed the experiment. RS, WL, and YT performed the laboratorial measurement and data analysis. WD and CH managed the field experiment. All authors discussed the results, and read and approved the final version of the manuscript. RS, WL, and BL wrote the paper.

## Conflict of Interest Statement

The authors declare that the research was conducted in the absence of any commercial or financial relationships that could be construed as a potential conflict of interest.
