# Supplementary material for: Tillage Changes Vertical Distribution of Soil Bacterial and Fungal Communities
Source: Front Microbiol. 2018 Apr 9;9:699. doi: 10.3389/fmicb.2018.00699 (PMC5900040; doi:10.3389/fmicb.2018.00699)
Supplement: Supplementary file 2 [file Table_2.DOCX]

**Table S2** P-value from the comparison of the linear regression slopes between microbial similarity and soil depth

| Soil property | Treatment | CT | RT | NT |
| --- | --- | --- | --- | --- |
| TC | CT |  | <0.001** | V<.001** |
|  | RT | <0.001** |  | 0.556 |
|  | NT | <0.001** | 0.556 |  |
| TN | CT |  | 0.003** | 0.049* |
|  | RT | 0.003** |  | 0.498 |
|  | NT | 0.049* | 0.498 |  |

CT, conventional plowing tillage; RT, rotary tillage; NT, no tillage.

TC, total carbon; TN, total carbon.

* represents significant difference: **, *P*<0.01; *, *P*<0.05.
