# Supplementary material for: Tillage Changes Vertical Distribution of Soil Bacterial and Fungal Communities
Source: Front Microbiol. 2018 Apr 9;9:699. doi: 10.3389/fmicb.2018.00699 (PMC5900040; doi:10.3389/fmicb.2018.00699)
Supplement: Supplementary file 3 [file Table_3.DOCX]

**Table S3** Contribution of soil depth and tillage to the variance of soil properties, bacterial and fungal communities determined by canonical variation partitioning

|  | Variance explained by (%) | |
| --- | --- | --- |
|  | Soil depth | Tillage |
| Soil properties | 36 | 1 |
| Bacterial richness | 14 | <1 |
| Bacterial community structure | 37 | 3 |
| Fungal richness | 23 | 29 |
| Fungal community structure | 10 | 6 |
