# Supplementary material for: Tillage Changes Vertical Distribution of Soil Bacterial and Fungal Communities
Source: Front Microbiol. 2018 Apr 9;9:699. doi: 10.3389/fmicb.2018.00699 (PMC5900040; doi:10.3389/fmicb.2018.00699)
Supplement: Supplementary file 4 [file Table_4.DOCX]

**Table S4** Abundance-based β-null deviation values for bacterial and fungal community calculated based on Bray-Curtis dissimilarity

| Soil layer | CT | |  | RT | | |  | NT | |
| --- | --- | --- | --- | --- | --- | --- | --- | --- | --- |
|  | Bacteria | Fungi |  | Bacteria | Fungi | |  | Bacteria | Fungi |
| 0-5cm | **0.62±0.006** | **0.55±0.013** |  | **0.62±0.009** | | **0.57±0.032** |  | **0.65±0.01** | **0.57±0.023** |
| 5-10cm | **0.60±0.006** | **0.54±0.023** |  | **0.60±0.005** | | **0.54±0.030** |  | **0.61±0.007** | **0.51±0.012** |
| 10-20cm | **0.60±0.003** | **0.51±0.001** |  | **0.60±0.003** | | **0.51±0.014** |  | **0.61±0.009** | **0.56±0.005** |
| 20-30cm | **0.63±0.007** | **0.55±0.026** |  | 0.55±0.004 | | 0.57±0.046 |  | **0.64±0.013** | **0.59±0.027** |

Shown are the mean ± standard deviation.

Bold fonts indicate significant difference between bacterial and fungal community (*P*<0.05) under same tillage treatment.
