# Supplementary material for: Tillage Changes Vertical Distribution of Soil Bacterial and Fungal Communities
Source: Front Microbiol. 2018 Apr 9;9:699. doi: 10.3389/fmicb.2018.00699 (PMC5900040; doi:10.3389/fmicb.2018.00699)
Supplement: Supplementary file 5 [file Image_1.PDF]

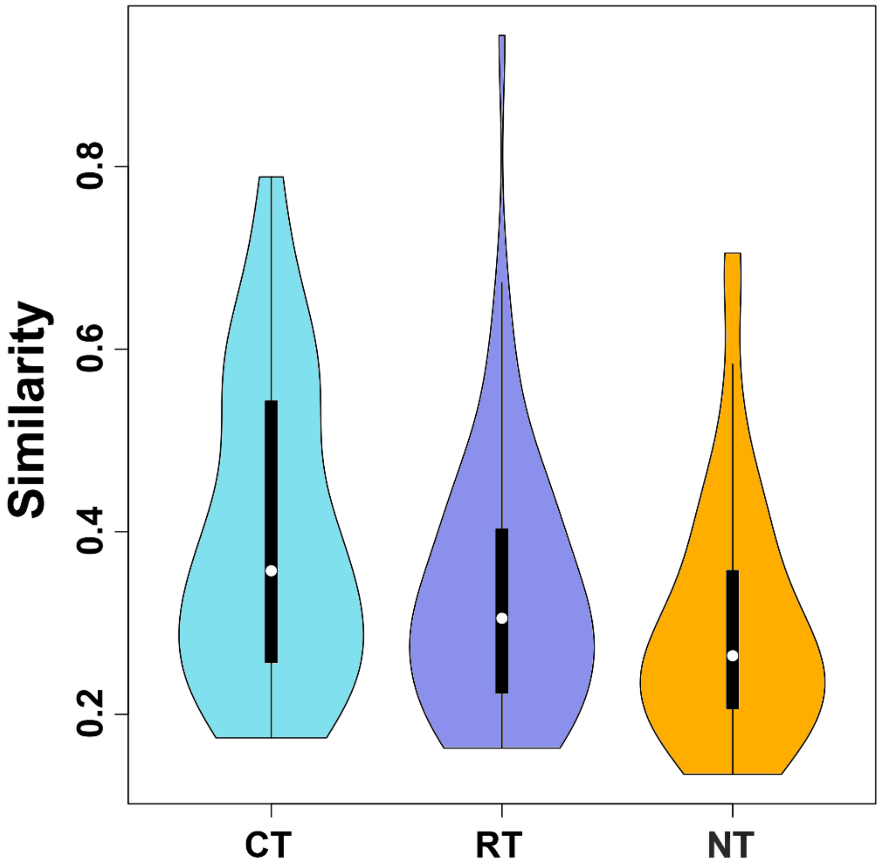

Figure S1. Violin plot showing the similarity of soil properties between soil layers under different tillage methods (CT, conventional plowing tillage; RT: rotary tillage; NT: no tillage)
The similarity was evaluated with the reciprocal of Euclidean distance.
