# Supplementary material for: Tillage Changes Vertical Distribution of Soil Bacterial and Fungal Communities
Source: Front Microbiol. 2018 Apr 9;9:699. doi: 10.3389/fmicb.2018.00699 (PMC5900040; doi:10.3389/fmicb.2018.00699)
Supplement: Supplementary file 6 [file Image_2.PDF]

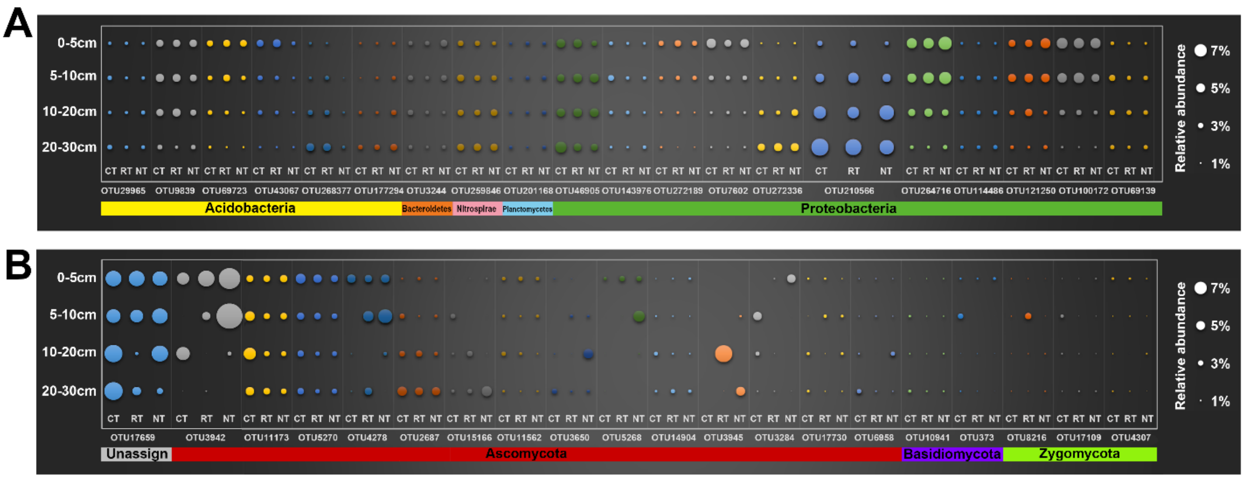

Figure S2. Dominant bacterial (A) and fungal (B) OTUs at different soil depths and under different tillage regimes
