# Supplementary material for: Tillage Changes Vertical Distribution of Soil Bacterial and Fungal Communities
Source: Front Microbiol. 2018 Apr 9;9:699. doi: 10.3389/fmicb.2018.00699 (PMC5900040; doi:10.3389/fmicb.2018.00699)
Supplement: Supplementary file 7 [file Image_3.PDF]

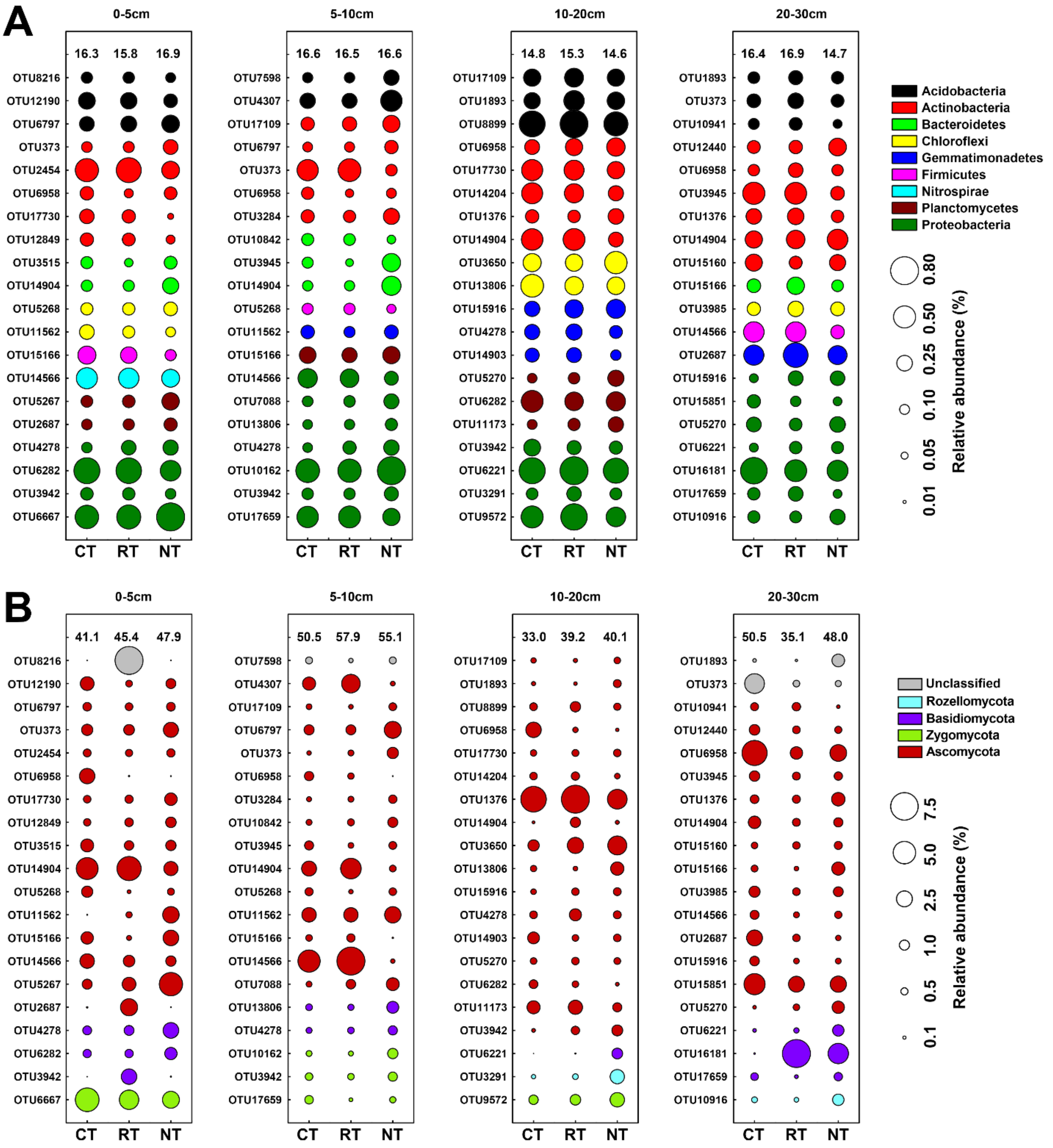

Figure S3. Bacterial (A) and fungal (B) OTUs that significantly changed in relative abundance between three tillage regimes.

Only the top 20 OTUs in relative abundance were present. The number at the top of each column showing the sum of relative abundance of OTUs that significantly changed between different tillage regimes.
