# Supplementary material for: Tillage Changes Vertical Distribution of Soil Bacterial and Fungal Communities
Source: Front Microbiol. 2018 Apr 9;9:699. doi: 10.3389/fmicb.2018.00699 (PMC5900040; doi:10.3389/fmicb.2018.00699)
Supplement: Supplementary file 8 [file Image_4.PDF]

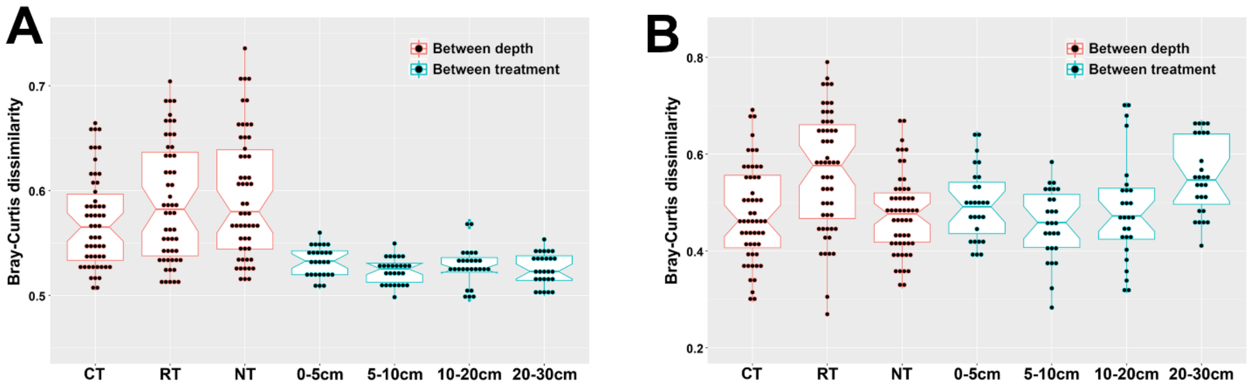


**Figure S4**. Bray-Curtis dissimilarity of bacterial (A) and fungal (B) communities between soil layers and different tillage treatments.
