# Supplementary material for: Tillage Changes Vertical Distribution of Soil Bacterial and Fungal Communities
Source: Front Microbiol. 2018 Apr 9;9:699. doi: 10.3389/fmicb.2018.00699 (PMC5900040; doi:10.3389/fmicb.2018.00699)
Supplement: Supplementary file 9 [file Image_5.PDF]

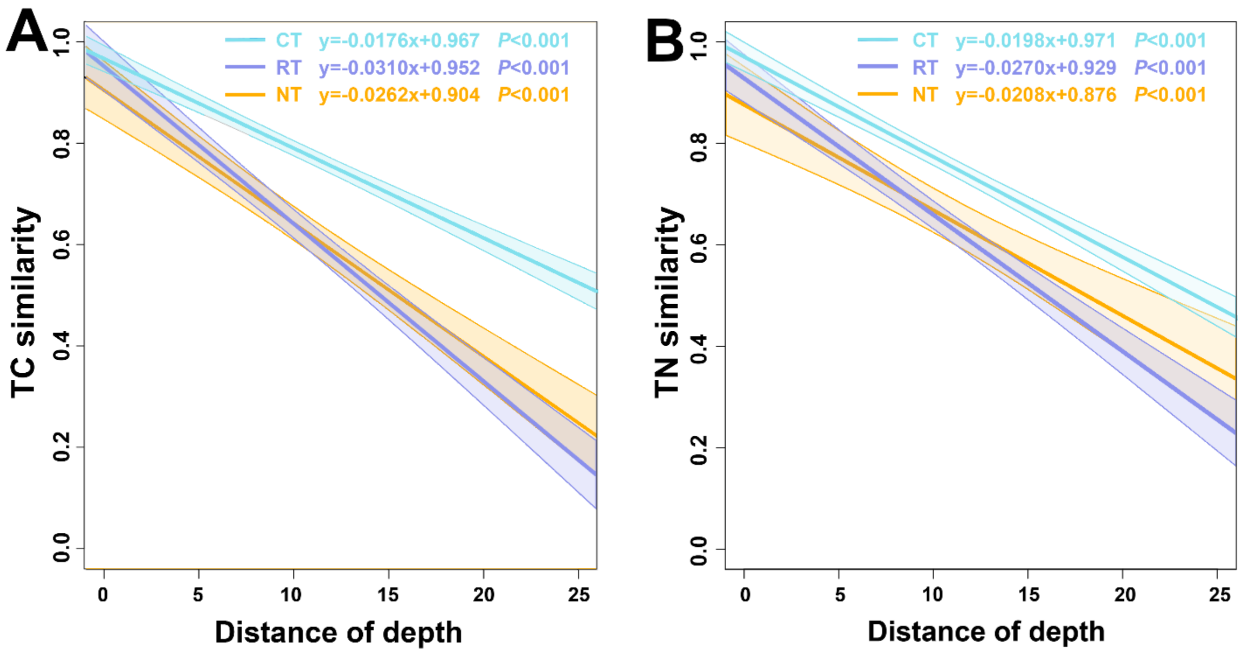


**Figure S5**. The depth-decay of similarity for total carbon (TC) (A) and total nitrogen (TN) (B) under different tillage practices.

CT, conventional plowing tillage; RT, rotary tillage; NT, no tillage.

The similarity was evaluated with 1-Gower distance.
