# Supplementary material for: Tillage Changes Vertical Distribution of Soil Bacterial and Fungal Communities
Source: Front Microbiol. 2018 Apr 9;9:699. doi: 10.3389/fmicb.2018.00699 (PMC5900040; doi:10.3389/fmicb.2018.00699)
Supplement: Supplementary file 10 [file Image_6.PDF]

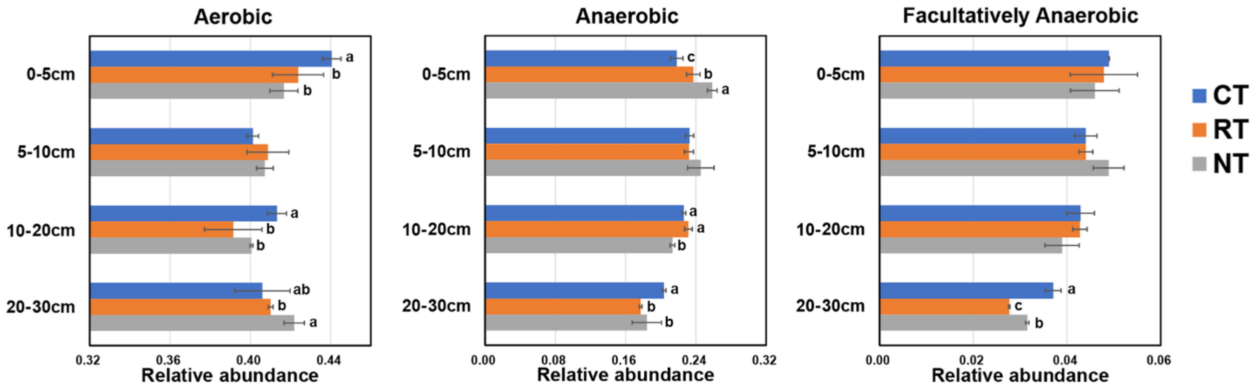

Figure S6. Relative abundance of bacterial groups with different preference for oxygen predicted by BugBase.

CT, conventional plowing tillage; RT, rotary tillage; NT, no tillage.

Bars with same letters indicate no significantly difference
